# A state level analyses of suicide and the COVID-19 pandemic in Mexico

**DOI:** 10.1186/s12888-022-04095-8

**Published:** 2022-07-09

**Authors:** G. Borges, J. A. Garcia, J. Pirkis, M. J. Spittal, D. Gunnell, M. Sinyor, A. John

**Affiliations:** 1 Instituto Nacional de Psiquiatría Ramon de La Fuente Muñiz, Calzada Mexico Xochimilco 101, 14370 Mexico City, CP Mexico; 2 Centro de Investigación Y Docencia Económicas (CIDE), Mexico City, Mexico; 3 Centre for Mental Health, Melbourne School of Population and Global Health, University of Melbourne, Melbourne, VIC Australia; 4 Population Health Sciences, Bristol Medical School, University of Bristol, Canynge Hall, Bristol, UK; 5 National Institute of Health Research Biomedical Research Centre at the University Hospitals Bristol and Weston NHS Foundation Trust and the University of Bristol, Bristol, UK; 6 Department of Psychiatry, Sunnybrook Health Sciences Centre, Toronto, Canada; 7 Department of Psychiatry, University of Toronto, Toronto, Canada; 8 Population Psychiatry, Suicide and Informatics, Medical School, Swansea University, Swansea, UK

**Keywords:** Suicide, Mexico, COVID-19, Epidemiology

## Abstract

**Background:**

While suicide rates in high- and middle-income countries appeared stable in the early stages of the pandemic, we know little about within-country variations. We sought to investigate the impact of COVID-19 on suicide in Mexico’s 32 states and to identify factors that may have contributed to observed variations between states.

**Methods:**

Interrupted time-series analysis to model the trend in monthly suicides before COVID-19 (from Jan 1, 2010, to March 31, 2020), comparing the expected number of suicides derived from the model with the observed number for the remainder of the year (April 1 to December 31, 2020) for each of Mexico’s 32 states. Next, we modeled state-level trends using linear regression to study likely contributing factors at ecological level.

**Results:**

Suicide increased slightly across Mexico during the first nine months of the pandemic (RR 1.03; 95%CI 1.01–1.05). Suicides remained stable in 19 states, increase in seven states (RR range: 1.12–2.04) and a decrease in six states (RR range: 0.46–0.88). Suicide RR at the state level was positively associated with population density in 2020 and state level suicide death rate in 2019.

**Conclusions:**

The COVID-19 pandemic had a differential effect on suicide death within the 32 states of Mexico. Higher population density and higher suicide rates in 2019 were associated with increased suicide. As the country struggles to cope with the ongoing pandemic, efforts to improve access to primary care and mental health care services (including suicide crisis intervention services) in these settings should be given priority.

**Supplementary Information:**

The online version contains supplementary material available at 10.1186/s12888-022-04095-8.

## Introduction

Globally, the COVID-19 pandemic has been characterized by wide variation in the geographical distribution of SARS Cov-2 infections and resulting disease, with several waves that have affected different countries at different times [1]. The worldwide variation is dynamic, and it is clear now that, within countries, there has also been a large variation in rates of COVID-19 across geographical and administrative divisions. These variations, in turn, have probably had a myriad of effects across a variety of health outcomes.

One such outcome with important public health implications is suicide. Because of the impact of the pandemic on known risk factors for suicide, such as unemployment, marginalization and population density [2] initially it was speculated that the pandemic might result in increases in suicides. However, our group’s analysis of suicide data from 21 countries/areas-within-countries, including Mexico City, in the early months of the pandemic concluded that “there does not appear to have been an increase in suicides since the pandemic began” with decreases in suicides after the pandemic in 12 countries or areas, relative risk (RR) range: 0.49–0.94) [3]. Although this study did include data at the state/provincial level for several countries, a comprehensive investigation of potential within-country variations was beyond its scope. A subsequent study examined local level (prefecture/city) geographical variations in suicide rates for one of the participating countries (Japan) [4]. It found that increased suicide rates at the prefecture/city-level during the pandemic were inversely related to rates of suicide from the prior year. That is, only cities that previously had low suicide rates showed an increase during the pandemic (incidence rate ratio (IRR) = 1.37 in the second outbreak); no association was found with confirmed COVID cases per million population, income, and urban population variations [4]. Another study examining geographical variations in hospitalizations for self-harm in France showed a weak negative correlation with hospitalization rates for COVID-19 with (Spearman’s rho = -0.21; *p* = 0.03) [5]. While new literature reviews summarized potential risk factors for changes in suicide during the pandemic [6, 7] we know few about ecological determinants of suicide that could be linked to the COVID-109 pandemic.

The dearth of studies that have rigorously investigated differences in the impact of COVID-19 on suicide within countries and possible factors associated with those differences is a substantial gap. To the best of our knowledge, there has been no report combining both elements to characterize suicide trends, before and during the pandemic, within large administrative/geographical units of a single country. This study addresses that gap through an in-depth analysis of the COVID-19 pandemic effect on suicide in the 32 states of Mexico, a country with almost 129 million inhabitants. On March 30, 2020, non-essential activities were suspended in Mexico because of the COVID-19 pandemic, and on March 31 a national emergency was declared. By December 31, 2020, Mexico had a large incidence of COVID-19 infection with 1.4 million cases and over 140,000 deaths (https://www.worldometers.info/coronavirus/country/mexico/). The goals of this study are to follow-up on the potential role of the COVID-19 pandemic on suicide trends in each of the 32 states of Mexico and to ecologically delve into alleged determinants of these trends.

## Methods

### Suicide trends

#### Study design

This is an interrupted time-series analysis that model trends in monthly suicides before COVID-19 (1 January 2010, to 31 March 2020), comparing the expected number of suicides with the observed number of suicides for the remainder of 2020 (1 April 2020 to 31 December 2020). We used this approach to obtain estimates of the association between the beginning of the pandemic on the suicide trends nationally for Mexico and for each of its 32 main geographical components (States)”.

#### Data sources

We sourced monthly suicide counts at the state level for Mexico (32 states, including State of Mexico with almost 17 million inhabitants and Colima with 731 thousand inhabitants) from cause of death records held by the main Mexican Statistical Bureau (INEGI) (http://www.dgis.salud.gob.mx/contenidos/basesdedatos/BD_Cubos_gobmx.html) that included the period from January 1, 2010, to December 31, 2020. Suicides were identified by the ICD-10 codes X60-X84. Our analysis used publicly available data without any individual identifiers. The full dataset is included in Appendix 1-dataset.

#### Statistical analyses for suicide trends

We plotted trends over time for the entire country and for each of its 32 states, along with a moving average filter (averaged over the prior two months, the current month and following two months). As with our group’s recent international study [3], we used time-series analysis with adjustments for time trends and seasonality to model the trends in monthly suicides from January 1, 2010, to March 31, 2020, before COVID-19. This pre-pandemic model was then used to forecast what the trend in suicides from the beginning of the COVID-19 period (April 1, 2020- December 31, 2020) would have been had the pandemic not happened. This calculation of the expected number of suicides based on the prior trend was compared with the observed number of suicides during the pandemic period by calculating a rate ratio (RR) and its 95% confidence interval. We followed this modeling strategy for time-series of the total suicides in Mexico and the suicides for each state.

Models were fitted using Poisson regression and accounted for possible over-dispersion using a scale parameter set to the model’s χ2 value divided by the residual degrees of freedom. We modelled the effect of time as a non-linear predictor. Non-linear time trends were estimated by selecting the best fitting model from a series of fractional polynomial models. Seasonality was accounted for with Fourier terms (pairs of sine and cosine functions) [8].

### Ecological level analyses

#### Study design

This is a multi-group ecological study for 32 states in Mexico. Here we study the impact of ecological risk factors (such as, for example, suicide death rates in each state in 2019) on the suicide trend for each state obtained from the prior analyses.

#### Data sources

Predictor variables at state-level that could help explain suicide trends during the first nine months of the pandemic across the country were selected based on prior reports of area-level predictors of self-harm and suicide [4, 5]. The full list of variables with a short description and sources of data for the ecological analyses is provided in Annex 1-Table 1.

**Table 1:** Bivariate and multiple linear model of the (log) rate ratio of suicide at State level (*n* = 32) against demographic, COVID-19 and suicide related variables, Mexico 2020

	**Bivariate**			**Multivariate**		
Variable	coefficient	*P*	95% CI	coefficient	*P*	95% CI
1-Unemployment III trimester 2020	0.0342266	0.13	-.0106754 .0791286	0.0210529	0.321	-.0217392 .063845
2-Population density 2020	0.000126	0.001	.0000571 .0001948	0.0001926	0.001	.000082 .0003033
3-Marginalization Index 2020	0.0008433	0.959	-.0320539 .0337406	-0.0247754	0.149	-.0590602 .0095094
4-COVID death rate	0.0012583	0.068	-.0001016 .0026181	-0.0010808	0.292	-.0031484 .0009867
5-Suicide in 2019 death rate	0.0119363	0.428	-.0183704 .042243	0.0284363	0.039	.0015273 .0553453

For a full description of variables see Table Supplementary 1

*COVID* Coronavirus disease 2019

#### Statistical analyses for ecological analyses

We used the RR estimates from the prior models to inspect variation in suicide trends in each state in Mexico. The log RR for each state was used as the outcome variable for an ecological analysis. As described in Annex 1- Table 1, they were general demographic and social data (population density, marginalization and unemployment), data related to direct effects of the pandemic (COVID-19 death rates) and pre-pandemic suicide rates at state level (from 2019).

We used the log RR calculated for each state as the outcome variable in a series of unadjusted and adjusted linear regressions to better understand the likely determinants of the RR distribution across the country. For graphical purposes, we divided states into those where there was statistical evidence of fewer than the predicted number of suicides during the pandemic era, based on previous trends (RR < 1), those with higher than predicted RRs (RR > 1) and those where there was no statistical evidence of a change in rate. (RR that include the null value in their confidence intervals).

All analyses were performed using Stata [9].

## Results

**Fig. 1:**
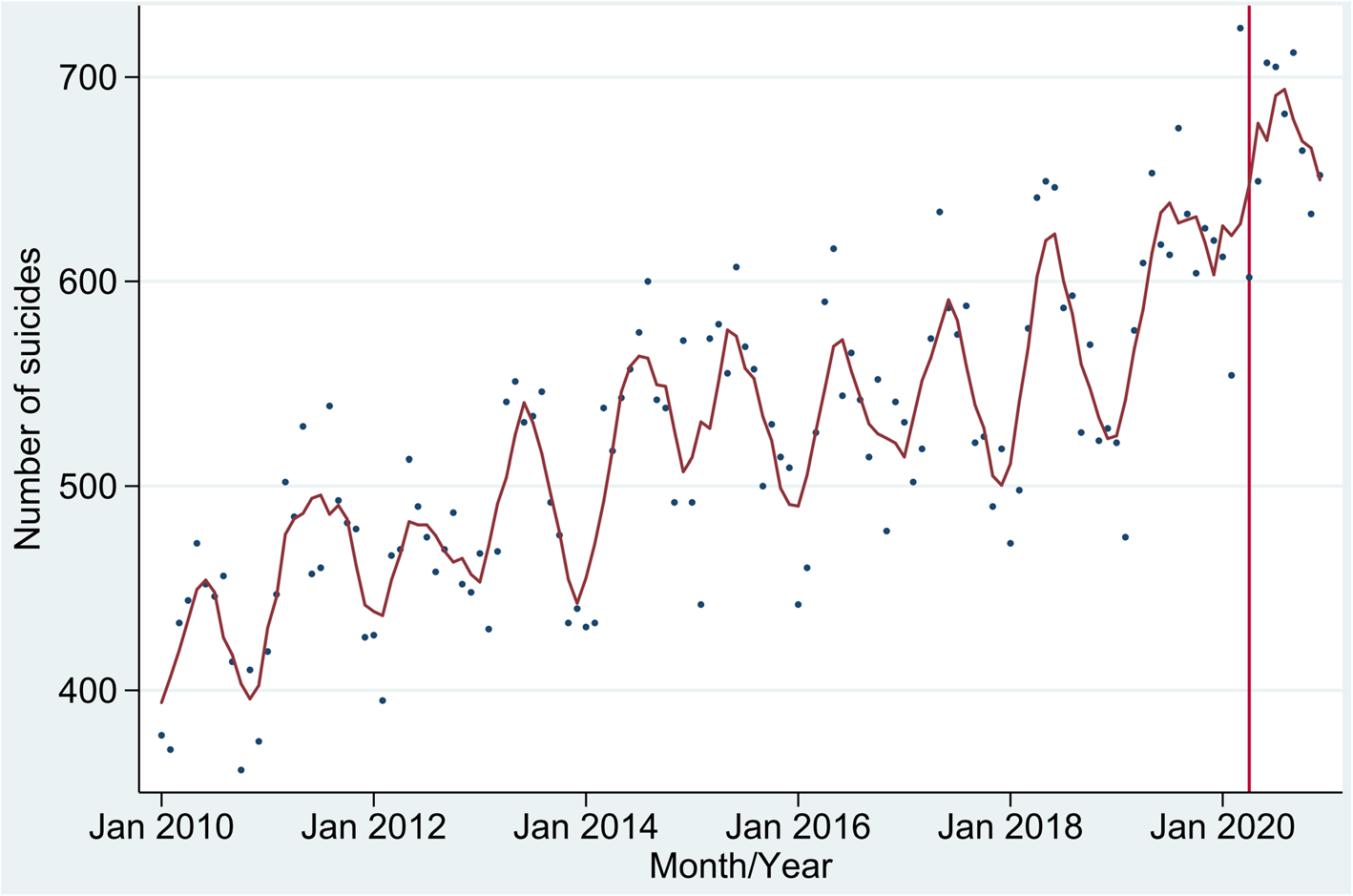
Time series plots of monthly suicides in Mexico

There were 7,826 suicides in Mexico during 2020, 6,006 of which occurred on or after April 1 during the pandemic period. As in prior years in Mexico, 81.7% of total suicides during 2020 occurred among males and 73.8% among those aged 45 or younger. Suicides in Mexico have been increasing during the past two decades and, as apparent in Fig. 1, seasonality also has an impact. The overall trend for the country, however, reflects a summation of diverse trends at the state level with some states showing decreases in suicides over time (e.g., Tabasco and Mexico City), others showing more stable patterns (e.g., Tamaulipas), and still others showing increases (e.g., Morelos) (see Annex2- Figure 1).

**Fig. 2:**
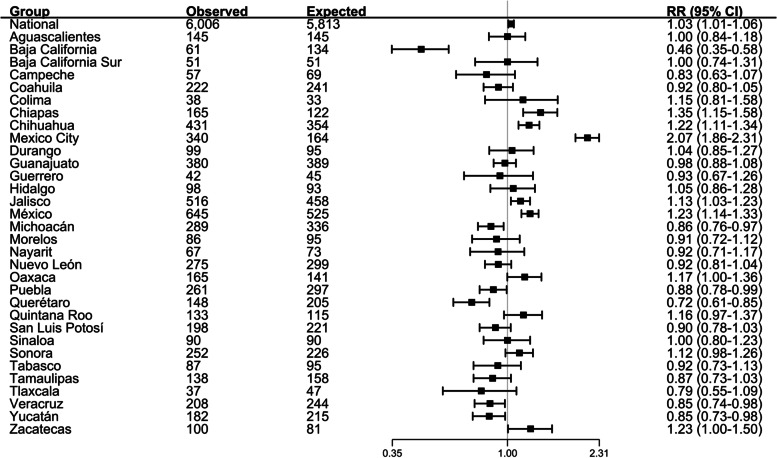
Observed and expected number of suicides, rate ratios (RR) and 95% confidence intervals (CI), 32 States in Mexico]

Based on suicide in prior years, our model predicted 5,813 suicides in Mexico during the nine months of the pandemic in 2020 (April-December). The 6,006 observed deaths correspond to a RR of 1.03 (95% CI 1.01–1.06), that is 3% more suicides than predicted based on trends at the national level (Fig. 2).

**Fig. 3:**
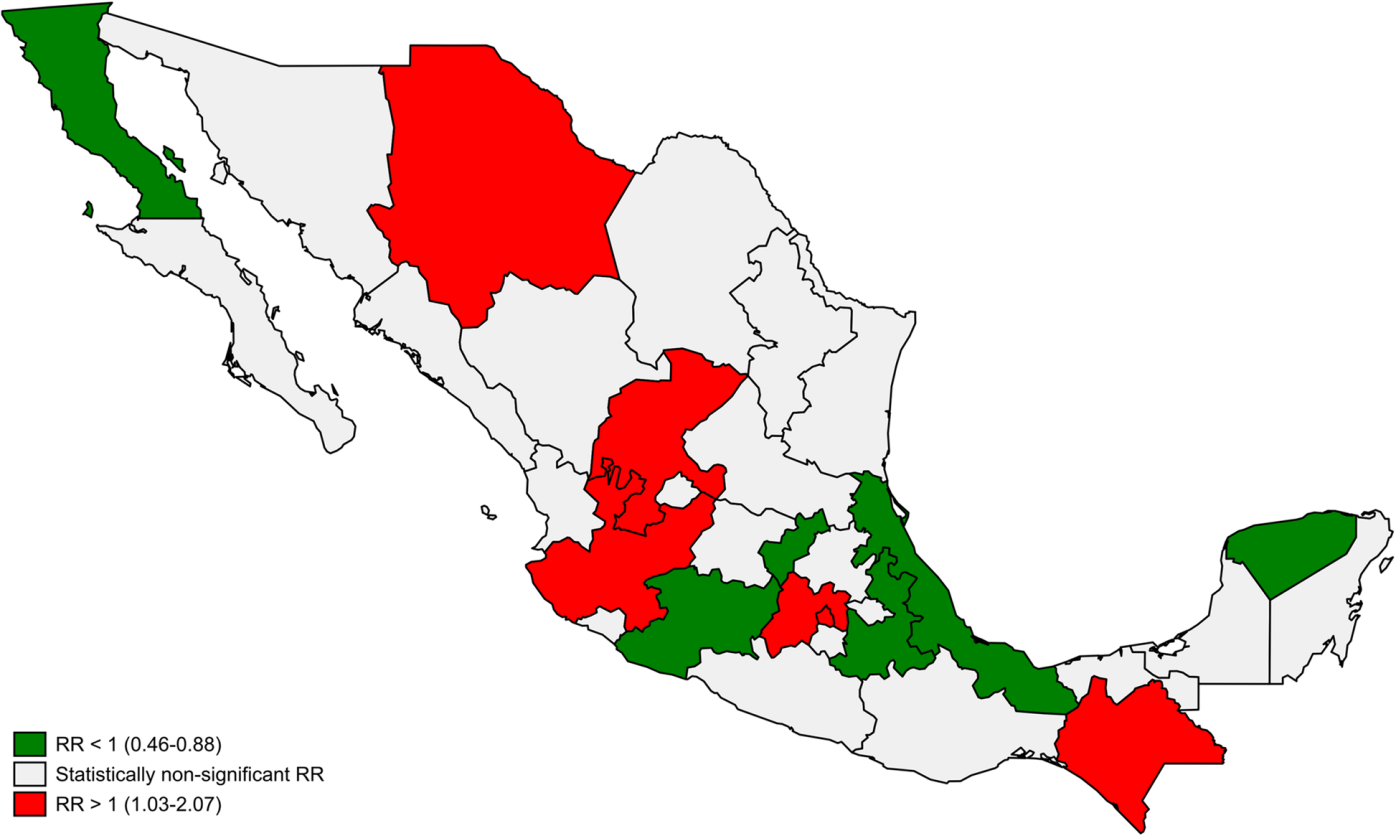
Suicide rate ratios in COVID-19 period (April to December 2020) compared to the trend observed in pre-COVID-19 period (January 2010-March 2020). Source: Own calculations with data from the National Institute of Statistics and Geography (INEGI), 2021 Rate ratio (RR) in national level was 1.03 (IC95 = 1.01–1.06)

This overall RR ranged from a low RR of 0.46 (95% CI 0.35–0.58) in Baja California to a high RR of 2.07 (95% CI 1.86–2.31) in Mexico City. A map of the country (Fig. 3) shows states with lower-than-expected rates (six states in green), higher than expected rates (seven states in red) and stable trends (19 states in grey). States in red and green are scattered across the country without any striking pattern.

We further inspected the possible role that general demographic and social variables, data related to direct effects of the pandemic (COVID-19 death rates) and suicide rates at state level in 2019 may have had on the distribution of these RRs across the country. In bivariate analyses, as shown in Table 1, population density in 2020 was positively associated with the RR distribution in linear regressions.

Next, we fitted a multiple regression with all pre-specified variables (Table 1). In this model, population density in 2020, and suicide rates at state level in 2019 were positively associated with the log RR distribution.

## Discussion

We found a small (3%) increase in suicides during the first nine months of the COVID-19 pandemic in Mexico encompassing diverse patterns of suicide across its 32 states. Notably, about 60% of states showed no change in suicide rates during the first months of the pandemic. No clear geographical pattern emerges from a visual inspection of these trends; however, we did identify that population density in year 2020, and suicide rates at state level in 2019 were positively associated with the distribution of RRs in states across Mexico.

The small increase in suicide we observed in Mexico during the first nine months of the pandemic is largely consistent with what we have reported previously regarding Mexico [3]; but here we have examined a larger number of months into the pandemic and data for the whole country, whereas previously only data from Mexico City were available. Our study identified RRs in Mexican states ranging from a low of 0.46 to a high of 2.07, underscoring that the impact of the pandemic on suicide rates should not be assumed to be uniform within countries. More recent studies reporting suicide rates internationally during the pandemic indicate a complex picture [10, 11], with some studies finding no change in suicide trends [12], others observing increasing trends [13] and still others identifying decreasing trends [14].

We further explored several factors that may explain these diverse trends, including basic demographic data and pre-pandemic suicide in the Mexican states. While we found that greater population density was associated with larger RRs of suicide in the pandemic period, less consistent results or even inverse relationships have been reported for this variable in other countries [15–17]. The mechanisms by which population density affects suicide may be different for males and females, may vary by age groups and may also vary by period [15, 17]. The possible role that population density may play in increased RR of suicide during the pandemic in Mexico could be multiple. Higher density is partially accounted for by overcrowded cities (Mexico City being an extreme example) and includes more urban environments, in general, which are often characterized by more limited or cramped living spaces. Such environments may have been particularly stressful in the context of a generational infectious disease outbreak and accompanying lockdown measures. Residents of cities may also adhere to more non-traditional family roles and experience less family cohesion. On the other hand, mental health resources in Mexico are much more available in urban areas [18]. Although we were not able to identify specific mechanisms by which population density acts on the RR of suicide with our current data, this study suggests rich material for future work in Mexico.

The finding that the states in Mexico with higher pre-pandemic suicide rates were more affected contrasts with Japan where suicide rates at the prefecture/city-level were inversely related to changes in suicide in the pandemic period [4]. In keeping with findings from Japan, in our adjusted analysis we found no evidence of an association with COVID mortality, although Tanaka’s analysis was based on COVID-19 incidence rather than mortality rates. Possible reasons for these different associations are largely speculative now. With a roughly similar number of habitants in 2020, the impact of COVID-19 was much larger in Mexico (144,000 deaths at 31, December 2020, versus 3,414 in Japan) than in Japan https://www.worldometers.info/coronavirus/#countries (https://www.worldometers.info/coronavirus/#countries). Japan and Mexico already had very different pre-pandemic rates of suicide, more than double in Japan (adjusted rate of 12.2 per 100,000 in 2019) compared to Mexico (5.3 per 100,000) [19], and trends of suicide in these two countries have been opposite in recent years, decreasing in Japan and increasing in Mexico. Pre-existing suicide rates may reflect underlying rates of mental illness, availability of means, and/or social attitudes toward suicide, and access to mental health and crisis care, all of which could also have contributed to suicide during the pandemic. We did observe that suicide has very different trends within Mexico itself and ours is the first study to identify higher rates in the immediate past as a potential marker of risk for more suicides during the pandemic. While this finding suggests possible avenues for targeted preventive efforts, focused on those areas with already high rates of suicide, much more research, spanning different regions and countries, is needed.

A recent review [10] discussed the complexities of the pandemic's impact on suicide and the nuances of this relationship that are likely to evolve over time and, we may now add, over space. Decisive actions by governments and society as a whole are nevertheless needed. As Mexico struggles to deal with the pandemic, we need to expand primary health care services and include suicide prevention and treatment in primary care settings [18]. Although the overall results of this study suggest that in most states in Mexico the pandemic had no net effects or was even associated with reductions in suicide mortality, this should not lead to complacency regarding effects in sub-populations or in the states that showed increase trends. As the pandemic continues to evolve, so there is a need to monitor and not be complacent in all states.

### Limitations

This study covers the first nine months of the COVID-19 pandemic in Mexico. It is possible that overall and state-specific trends may change as the pandemic evolves and the country faces the daunting challenges of economic disruption. Follow-up studies into 2021 and beyond should be carried out and, most importantly, evaluation of the policies used to address economic downturn and manage mental health issues and suicidality are needed. While most states in Mexico are large, overall suicide rates are relatively low by international standards [20] and some of our analyses are based on small number of suicides in some states, as in the three states there were fewer than 50 suicides in 2020. Our data on suicide is limited by the current stage of the health system statics in Mexico, and improvements in the quality of death certificates in the country during the period may have had an impact on the results. Our ecological analyses of factors that potentially contributed to the RR distribution were based on 32 states, therefore, low power may explain the lack of associations with some of our predictor variables. We did not take account of spatial autocorrelation in the ecological analysis, but this is unlikely to have had a major impact on our findings. These results indicate associations and as in any other ecological study it is not possible to infer causality from our results. The discrepancies found between studies should be used as a starting point for more in-depth inquiry into causal mechanisms, with more astringent methodologies beyond ecological level studies. Advances in the enquire of the role of the COVID-19 pandemic on suicide across regional areas could then lead to formal theories that, in turn, could advance the formulation of hypothesis to be tested. At present, our knowledge is quite limited to the report of seemingly discrepant findings.

## Conclusions

Large differences were found at the state level on the impact of COVID-19 in suicide rates, and much more research is needed to confirm and further characterize the role of key variables in this variation. While our results cannot establish causality, they support the notion that targeted suicide prevention efforts in areas of higher population density and higher pre-existing suicide rates might help to mitigate negative effects of pandemics on suicide.

## Supplementary Information

**Additional file 1: Table 1.** Description and sources of variables used for correlations with log rr of suicide during 9 months into the pandemic.

**Additional file 2: Figure 1.** Time series plot of monthly suicides in Mexico for each state.

**Additional file 3: Appendix S1.** Suicide data set.

## Data Availability

The data underlying this article were accessed from National Institute of Statistics and Geography, Mexico (https://www.inegi.org.mx/temas/mortalidad/). The derived data generated in this research will be shared on reasonable request to the corresponding author. The full dataset for suicide mortality is included in Appendix 1-dataset.
