# Supplementary material for: A state level analyses of suicide and the COVID-19 pandemic in Mexico
Source: BMC Psychiatry. 2022 Jul 9;22:460. doi: 10.1186/s12888-022-04095-8 (PMC9271255; doi:10.1186/s12888-022-04095-8)
Supplement: Supplementary file 1 — Additional file 1: Table 1. Description and sources of variables used for correlations with log rr of suicide during 9 months into the pandemic. [file 12888_2022_4095_MOESM1_ESM.docx]

| **Annex Table 1- Description and sources of variables used for correlations with log rr of suicide during 9 months into the pandemic-** | |
| --- | --- |
| **Variable description** | **Source** |
| Unemployment III trimester in 2020: Percentage of unemployed population with respect to the Economically Active Population. Encuesta Nacional de Ocupación y Empleo (ENOE) data for the third quarter (July-September) at state level in 2020. | National Occupation and Employment Survey (ENOE), population 15 years of age and older. (https://www.inegi.org.mx/programas/enoe/15ymas/) |
| Population density in 2020: Data from the 2020 Population Census. Percentage of total population with respect to the territorial area of the state level in 2020. | Data is from 2020 census (https://www.inegi.org.mx/programas/ccpv/2020/default.html) |
| Marginalization Index in 2020: Index developed by the National Population Council (CONAPO) by geographic area. Measures the intensity of deprivation and social exclusion of the population according to the increases in value as higher percentages of the population live in small localities, in inadequate housing, with lack of access to education and low income at state level in 2020. | National Population Council (CONAPO) (https://www.gob.mx/conapo/documentos/indices-de-marginacion-2020-284372) |
| Death rate COVID-19 x 100000 inhabitants at state level in 2020 | Death rate from COVID-19 cases in 2020 is from governmental sources: CONACYT and Health Ministry (https://datos.covid-19.conacyt.mx/) |
| Suicide death rate x 100000 inhabitants at state level in 2019 | Suicide death rate in 2019 is from governmental sources: (http://www.dgis.salud.gob.mx/contenidos/basesdedatos/BD_Cubos_gobmx.html) |
