# Supplementary material for: A state level analyses of suicide and the COVID-19 pandemic in Mexico
Source: BMC Psychiatry. 2022 Jul 9;22:460. doi: 10.1186/s12888-022-04095-8 (PMC9271255; doi:10.1186/s12888-022-04095-8)
Supplement: Supplementary file 2 — Additional file 2: Figure 1. Time series plot of monthly suicides in Mexico for each state. [file 12888_2022_4095_MOESM2_ESM.docx]

Title: Annex 2- Figure 1 Time series plot of monthly suicides in Mexico for each state
