# Supplementary material for: A state level analyses of suicide and the COVID-19 pandemic in Mexico
Source: BMC Psychiatry. 2022 Jul 9;22:460. doi: 10.1186/s12888-022-04095-8 (PMC9271255; doi:10.1186/s12888-022-04095-8)
Supplement: Supplementary file 3 — Additional file 3: Appendix S1. Suicide data set. [file 12888_2022_4095_MOESM3_ESM.docx]

Supplementary Appendix S1- Suicide data set

National Suicide Data

|  | Jan | Feb | Mar | Apr | May | Jun | Jul | Aug | Sep | Oct | Nov | Dec |
| --- | --- | --- | --- | --- | --- | --- | --- | --- | --- | --- | --- | --- |
| 2010 | 378 | 371 | 433 | 444 | 472 | 452 | 446 | 456 | 414 | 361 | 410 | 375 |
| 2011 | 419 | 447 | 502 | 485 | 529 | 457 | 460 | 539 | 493 | 482 | 479 | 426 |
| 2012 | 427 | 395 | 466 | 469 | 513 | 490 | 475 | 458 | 469 | 487 | 452 | 448 |
| 2013 | 467 | 430 | 468 | 541 | 551 | 531 | 534 | 546 | 492 | 476 | 433 | 440 |
| 2014 | 431 | 433 | 538 | 517 | 543 | 557 | 575 | 600 | 542 | 538 | 492 | 571 |
| 2015 | 492 | 442 | 572 | 579 | 555 | 607 | 568 | 557 | 500 | 530 | 514 | 509 |
| 2016 | 442 | 460 | 526 | 590 | 616 | 544 | 565 | 542 | 514 | 552 | 478 | 541 |
| 2017 | 531 | 502 | 518 | 572 | 634 | 587 | 574 | 588 | 521 | 524 | 490 | 518 |
| 2018 | 472 | 498 | 577 | 641 | 649 | 646 | 587 | 593 | 526 | 569 | 522 | 528 |
| 2019 | 521 | 475 | 576 | 609 | 653 | 618 | 613 | 675 | 633 | 604 | 626 | 620 |
| 2020 | 612 | 554 | 724 | 602 | 649 | 707 | 705 | 682 | 712 | 664 | 633 | 652 |

Aguascalientes

|  | Jan | Feb | Mar | Apr | May | Jun | Jul | Aug | Sep | Oct | Nov | Dec |
| --- | --- | --- | --- | --- | --- | --- | --- | --- | --- | --- | --- | --- |
| 2010 | 5 | 1 | 2 | 4 | 11 | 4 | 5 | 4 | 4 | 4 | 3 | 3 |
| 2011 | 5 | 9 | 8 | 12 | 10 | 12 | 8 | 7 | 7 | 5 | 10 | 6 |
| 2012 | 6 | 7 | 6 | 14 | 15 | 10 | 9 | 5 | 15 | 10 | 11 | 6 |
| 2013 | 6 | 5 | 9 | 11 | 11 | 18 | 13 | 9 | 6 | 8 | 9 | 8 |
| 2014 | 3 | 8 | 7 | 11 | 12 | 14 | 9 | 8 | 9 | 5 | 10 | 13 |
| 2015 | 10 | 5 | 10 | 7 | 7 | 10 | 15 | 7 | 14 | 18 | 11 | 12 |
| 2016 | 7 | 6 | 15 | 8 | 15 | 9 | 10 | 8 | 12 | 9 | 9 | 17 |
| 2017 | 7 | 11 | 10 | 13 | 11 | 13 | 14 | 9 | 14 | 14 | 13 | 10 |
| 2018 | 10 | 8 | 14 | 16 | 16 | 13 | 8 | 13 | 10 | 12 | 13 | 7 |
| 2019 | 14 | 9 | 12 | 17 | 17 | 17 | 18 | 12 | 15 | 15 | 9 | 21 |
| 2020 | 20 | 9 | 10 | 15 | 19 | 16 | 20 | 13 | 14 | 9 | 17 | 22 |

Baja California

|  | Jan | Feb | Mar | Apr | May | Jun | Jul | Aug | Sep | Oct | Nov | Dec |
| --- | --- | --- | --- | --- | --- | --- | --- | --- | --- | --- | --- | --- |
| 2010 | 8 | 9 | 10 | 7 | 11 | 10 | 12 | 11 | 8 | 9 | 7 | 5 |
| 2011 | 5 | 16 | 12 | 7 | 8 | 18 | 12 | 16 | 16 | 11 | 10 | 14 |
| 2012 | 11 | 7 | 12 | 11 | 6 | 11 | 10 | 13 | 7 | 13 | 8 | 3 |
| 2013 | 6 | 4 | 8 | 7 | 9 | 5 | 11 | 17 | 18 | 14 | 15 | 7 |
| 2014 | 13 | 14 | 15 | 14 | 17 | 17 | 23 | 20 | 9 | 11 | 11 | 16 |
| 2015 | 13 | 7 | 9 | 14 | 15 | 14 | 19 | 7 | 17 | 12 | 16 | 9 |
| 2016 | 8 | 6 | 16 | 16 | 16 | 17 | 15 | 8 | 13 | 14 | 27 | 21 |
| 2017 | 17 | 12 | 17 | 14 | 17 | 10 | 20 | 13 | 22 | 21 | 12 | 14 |
| 2018 | 17 | 21 | 15 | 16 | 14 | 20 | 16 | 22 | 17 | 14 | 20 | 20 |
| 2019 | 18 | 8 | 11 | 8 | 10 | 25 | 20 | 18 | 22 | 12 | 15 | 12 |
| 2020 | 12 | 13 | 22 | 16 | 6 | 4 | 9 | 9 | 12 | 3 | 1 | 1 |

Baja California Sur

|  | Jan | Feb | Mar | Apr | May | Jun | Jul | Aug | Sep | Oct | Nov | Dec |
| --- | --- | --- | --- | --- | --- | --- | --- | --- | --- | --- | --- | --- |
| 2010 | 7 | 2 | 4 | 2 | 5 | 3 | 3 | 2 | 9 | 3 | 2 | 3 |
| 2011 | 6 | 2 | 3 | 1 | 0 | 4 | 1 | 4 | 3 | 2 | 1 | 2 |
| 2012 | 4 | 6 | 3 | 3 | 10 | 4 | 2 | 3 | 2 | 4 | 2 | 5 |
| 2013 | 7 | 4 | 3 | 8 | 6 | 2 | 4 | 5 | 4 | 4 | 1 | 2 |
| 2014 | 3 | 4 | 3 | 3 | 7 | 5 | 7 | 9 | 2 | 4 | 7 | 5 |
| 2015 | 2 | 2 | 6 | 5 | 6 | 6 | 6 | 7 | 6 | 3 | 5 | 3 |
| 2016 | 3 | 3 | 3 | 3 | 6 | 8 | 2 | 4 | 8 | 7 | 3 | 6 |
| 2017 | 2 | 3 | 4 | 3 | 3 | 5 | 4 | 4 | 5 | 8 | 1 | 6 |
| 2018 | 4 | 4 | 5 | 3 | 4 | 13 | 9 | 1 | 5 | 2 | 6 | 6 |
| 2019 | 4 | 8 | 9 | 6 | 2 | 5 | 6 | 6 | 3 | 4 | 4 | 5 |
| 2020 | 7 | 9 | 3 | 2 | 5 | 6 | 11 | 10 | 5 | 4 | 3 | 5 |

Campeche

|  | Jan | Feb | Mar | Apr | May | Jun | Jul | Aug | Sep | Oct | Nov | Dec |
| --- | --- | --- | --- | --- | --- | --- | --- | --- | --- | --- | --- | --- |
| 2010 | 0 | 0 | 6 | 6 | 10 | 7 | 4 | 5 | 8 | 6 | 5 | 5 |
| 2011 | 3 | 6 | 4 | 2 | 7 | 12 | 13 | 6 | 10 | 5 | 7 | 3 |
| 2012 | 4 | 6 | 8 | 7 | 6 | 5 | 5 | 4 | 8 | 3 | 3 | 3 |
| 2013 | 8 | 8 | 4 | 8 | 2 | 5 | 10 | 8 | 5 | 10 | 4 | 5 |
| 2014 | 4 | 6 | 6 | 7 | 6 | 15 | 8 | 5 | 8 | 9 | 4 | 12 |
| 2015 | 7 | 5 | 12 | 8 | 8 | 8 | 10 | 4 | 5 | 6 | 6 | 6 |
| 2016 | 5 | 7 | 5 | 3 | 7 | 7 | 8 | 5 | 16 | 7 | 7 | 9 |
| 2017 | 6 | 7 | 9 | 5 | 7 | 7 | 7 | 6 | 6 | 7 | 4 | 5 |
| 2018 | 6 | 8 | 2 | 7 | 8 | 6 | 5 | 8 | 5 | 8 | 6 | 3 |
| 2019 | 6 | 3 | 7 | 12 | 11 | 5 | 6 | 14 | 13 | 4 | 12 | 6 |
| 2020 | 6 | 3 | 6 | 5 | 3 | 7 | 7 | 5 | 8 | 4 | 10 | 8 |

Coahuila

|  | Jan | Feb | Mar | Apr | May | Jun | Jul | Aug | Sep | Oct | Nov | Dec |
| --- | --- | --- | --- | --- | --- | --- | --- | --- | --- | --- | --- | --- |
| 2010 | 9 | 15 | 22 | 9 | 20 | 15 | 21 | 19 | 12 | 14 | 4 | 10 |
| 2011 | 10 | 12 | 14 | 8 | 17 | 13 | 20 | 16 | 19 | 16 | 14 | 11 |
| 2012 | 14 | 14 | 7 | 15 | 12 | 15 | 17 | 11 | 15 | 11 | 9 | 12 |
| 2013 | 7 | 13 | 17 | 16 | 13 | 19 | 17 | 20 | 16 | 13 | 8 | 14 |
| 2014 | 14 | 9 | 15 | 13 | 16 | 16 | 14 | 14 | 14 | 21 | 11 | 19 |
| 2015 | 16 | 11 | 17 | 13 | 16 | 16 | 20 | 18 | 13 | 17 | 12 | 11 |
| 2016 | 10 | 12 | 16 | 16 | 20 | 14 | 17 | 11 | 10 | 16 | 8 | 18 |
| 2017 | 20 | 15 | 8 | 22 | 19 | 23 | 22 | 19 | 13 | 14 | 16 | 12 |
| 2018 | 14 | 15 | 16 | 29 | 17 | 22 | 19 | 28 | 16 | 13 | 17 | 20 |
| 2019 | 19 | 13 | 25 | 17 | 21 | 28 | 26 | 30 | 17 | 34 | 17 | 24 |
| 2020 | 16 | 18 | 19 | 17 | 19 | 35 | 17 | 28 | 32 | 25 | 22 | 27 |

Colima

|  | Jan | Feb | Mar | Apr | May | Jun | Jul | Aug | Sep | Oct | Nov | Dec |
| --- | --- | --- | --- | --- | --- | --- | --- | --- | --- | --- | --- | --- |
| 2010 | 3 | 4 | 0 | 5 | 1 | 3 | 2 | 3 | 3 | 2 | 3 | 1 |
| 2011 | 6 | 5 | 7 | 3 | 3 | 4 | 1 | 2 | 1 | 5 | 3 | 5 |
| 2012 | 2 | 4 | 5 | 6 | 7 | 5 | 8 | 2 | 3 | 1 | 4 | 3 |
| 2013 | 8 | 5 | 5 | 3 | 7 | 1 | 2 | 5 | 2 | 6 | 3 | 3 |
| 2014 | 4 | 4 | 2 | 5 | 3 | 4 | 3 | 6 | 4 | 3 | 2 | 3 |
| 2015 | 5 | 2 | 4 | 6 | 8 | 5 | 3 | 4 | 5 | 3 | 7 | 3 |
| 2016 | 5 | 7 | 9 | 9 | 4 | 7 | 7 | 4 | 5 | 3 | 6 | 1 |
| 2017 | 4 | 2 | 5 | 7 | 7 | 3 | 8 | 2 | 2 | 2 | 1 | 5 |
| 2018 | 8 | 6 | 3 | 5 | 3 | 3 | 4 | 4 | 4 | 5 | 5 | 3 |
| 2019 | 1 | 3 | 2 | 3 | 4 | 5 | 4 | 3 | 5 | 9 | 0 | 3 |
| 2020 | 6 | 6 | 5 | 2 | 4 | 6 | 4 | 1 | 4 | 3 | 4 | 10 |

Chiapas

|  | Jan | Feb | Mar | Apr | May | Jun | Jul | Aug | Sep | Oct | Nov | Dec |
| --- | --- | --- | --- | --- | --- | --- | --- | --- | --- | --- | --- | --- |
| 2010 | 3 | 1 | 7 | 5 | 11 | 6 | 7 | 8 | 6 | 7 | 5 | 19 |
| 2011 | 16 | 16 | 18 | 25 | 23 | 9 | 23 | 25 | 23 | 15 | 22 | 12 |
| 2012 | 11 | 7 | 5 | 10 | 14 | 15 | 13 | 19 | 21 | 29 | 17 | 26 |
| 2013 | 26 | 20 | 21 | 24 | 26 | 24 | 27 | 28 | 28 | 27 | 27 | 30 |
| 2014 | 18 | 26 | 28 | 19 | 28 | 20 | 27 | 43 | 22 | 32 | 22 | 34 |
| 2015 | 19 | 12 | 43 | 26 | 32 | 27 | 41 | 23 | 24 | 26 | 24 | 18 |
| 2016 | 6 | 24 | 34 | 22 | 33 | 24 | 22 | 28 | 10 | 19 | 21 | 27 |
| 2017 | 22 | 25 | 23 | 19 | 24 | 25 | 22 | 22 | 10 | 26 | 26 | 21 |
| 2018 | 8 | 11 | 22 | 21 | 19 | 16 | 23 | 20 | 11 | 16 | 14 | 16 |
| 2019 | 13 | 17 | 11 | 10 | 20 | 15 | 13 | 22 | 12 | 14 | 24 | 27 |
| 2020 | 11 | 10 | 22 | 24 | 18 | 13 | 18 | 14 | 19 | 21 | 16 | 22 |

Chihuahua

|  | Jan | Feb | Mar | Apr | May | Jun | Jul | Aug | Sep | Oct | Nov | Dec |
| --- | --- | --- | --- | --- | --- | --- | --- | --- | --- | --- | --- | --- |
| 2010 | 18 | 15 | 12 | 23 | 20 | 22 | 23 | 27 | 12 | 21 | 20 | 21 |
| 2011 | 15 | 21 | 26 | 22 | 29 | 31 | 29 | 34 | 19 | 21 | 21 | 24 |
| 2012 | 20 | 19 | 20 | 27 | 26 | 27 | 27 | 21 | 20 | 17 | 19 | 21 |
| 2013 | 20 | 22 | 25 | 31 | 30 | 33 | 33 | 22 | 27 | 25 | 28 | 17 |
| 2014 | 23 | 24 | 31 | 32 | 35 | 33 | 30 | 29 | 29 | 25 | 21 | 15 |
| 2015 | 31 | 29 | 26 | 51 | 37 | 42 | 37 | 40 | 42 | 28 | 29 | 30 |
| 2016 | 32 | 27 | 31 | 36 | 45 | 44 | 45 | 47 | 42 | 37 | 28 | 25 |
| 2017 | 40 | 31 | 25 | 42 | 33 | 36 | 53 | 41 | 32 | 34 | 18 | 25 |
| 2018 | 24 | 24 | 33 | 43 | 36 | 48 | 36 | 31 | 33 | 29 | 29 | 32 |
| 2019 | 19 | 32 | 42 | 33 | 33 | 49 | 36 | 45 | 46 | 34 | 38 | 40 |
| 2020 | 38 | 33 | 46 | 35 | 42 | 67 | 51 | 64 | 48 | 44 | 33 | 47 |

Mexico City

|  | Jan | Feb | Mar | Apr | May | Jun | Jul | Aug | Sep | Oct | Nov | Dec |
| --- | --- | --- | --- | --- | --- | --- | --- | --- | --- | --- | --- | --- |
| 2010 | 32 | 27 | 33 | 46 | 35 | 33 | 35 | 37 | 23 | 25 | 26 | 31 |
| 2011 | 33 | 44 | 25 | 39 | 41 | 19 | 27 | 33 | 49 | 37 | 39 | 38 |
| 2012 | 26 | 29 | 48 | 32 | 45 | 28 | 36 | 27 | 35 | 42 | 41 | 35 |
| 2013 | 38 | 37 | 31 | 34 | 38 | 43 | 30 | 35 | 43 | 32 | 36 | 34 |
| 2014 | 23 | 37 | 34 | 41 | 35 | 42 | 39 | 43 | 46 | 32 | 46 | 41 |
| 2015 | 34 | 30 | 38 | 35 | 33 | 38 | 23 | 30 | 20 | 31 | 32 | 29 |
| 2016 | 30 | 30 | 23 | 42 | 27 | 29 | 35 | 36 | 34 | 26 | 34 | 33 |
| 2017 | 26 | 29 | 16 | 39 | 34 | 23 | 22 | 19 | 18 | 27 | 17 | 31 |
| 2018 | 19 | 22 | 17 | 29 | 19 | 25 | 22 | 23 | 13 | 14 | 13 | 12 |
| 2019 | 7 | 13 | 10 | 8 | 16 | 19 | 9 | 17 | 31 | 28 | 37 | 31 |
| 2020 | 27 | 30 | 50 | 32 | 41 | 57 | 35 | 36 | 25 | 39 | 35 | 40 |

Durango

|  | Jan | Feb | Mar | Apr | May | Jun | Jul | Aug | Sep | Oct | Nov | Dec |
| --- | --- | --- | --- | --- | --- | --- | --- | --- | --- | --- | --- | --- |
| 2010 | 6 | 7 | 3 | 3 | 11 | 7 | 13 | 12 | 7 | 4 | 11 | 10 |
| 2011 | 5 | 11 | 6 | 8 | 4 | 6 | 8 | 8 | 11 | 5 | 6 | 5 |
| 2012 | 4 | 7 | 6 | 5 | 8 | 5 | 10 | 12 | 4 | 4 | 5 | 4 |
| 2013 | 8 | 7 | 8 | 10 | 6 | 9 | 9 | 7 | 9 | 4 | 9 | 4 |
| 2014 | 8 | 3 | 6 | 8 | 12 | 11 | 10 | 9 | 11 | 10 | 4 | 9 |
| 2015 | 8 | 6 | 9 | 12 | 14 | 8 | 11 | 11 | 11 | 8 | 8 | 7 |
| 2016 | 8 | 13 | 10 | 7 | 13 | 9 | 11 | 10 | 10 | 12 | 8 | 7 |
| 2017 | 6 | 12 | 10 | 4 | 16 | 16 | 15 | 7 | 6 | 13 | 14 | 7 |
| 2018 | 10 | 9 | 7 | 12 | 21 | 9 | 11 | 14 | 10 | 12 | 7 | 5 |
| 2019 | 6 | 5 | 8 | 18 | 12 | 14 | 16 | 13 | 12 | 9 | 10 | 14 |
| 2020 | 4 | 4 | 13 | 12 | 12 | 12 | 9 | 10 | 14 | 11 | 8 | 11 |

Guanajuato

|  | Jan | Feb | Mar | Apr | May | Jun | Jul | Aug | Sep | Oct | Nov | Dec |
| --- | --- | --- | --- | --- | --- | --- | --- | --- | --- | --- | --- | --- |
| 2010 | 15 | 19 | 30 | 21 | 20 | 31 | 27 | 32 | 24 | 13 | 27 | 25 |
| 2011 | 23 | 24 | 45 | 23 | 26 | 26 | 32 | 39 | 25 | 31 | 34 | 27 |
| 2012 | 26 | 25 | 28 | 22 | 32 | 42 | 26 | 23 | 23 | 33 | 30 | 29 |
| 2013 | 23 | 28 | 24 | 37 | 37 | 40 | 24 | 25 | 37 | 33 | 25 | 22 |
| 2014 | 27 | 23 | 36 | 36 | 35 | 43 | 40 | 39 | 33 | 38 | 36 | 38 |
| 2015 | 32 | 36 | 37 | 45 | 43 | 51 | 39 | 36 | 27 | 50 | 37 | 33 |
| 2016 | 33 | 26 | 43 | 46 | 49 | 38 | 39 | 31 | 26 | 47 | 32 | 43 |
| 2017 | 36 | 40 | 39 | 46 | 46 | 43 | 37 | 48 | 34 | 40 | 48 | 40 |
| 2018 | 36 | 28 | 57 | 53 | 44 | 49 | 40 | 48 | 43 | 42 | 42 | 32 |
| 2019 | 35 | 38 | 47 | 42 | 45 | 46 | 40 | 40 | 41 | 44 | 41 | 43 |
| 2020 | 41 | 41 | 40 | 51 | 37 | 54 | 45 | 33 | 50 | 41 | 36 | 33 |

Guerrero

|  | Jan | Feb | Mar | Apr | May | Jun | Jul | Aug | Sep | Oct | Nov | Dec |
| --- | --- | --- | --- | --- | --- | --- | --- | --- | --- | --- | --- | --- |
| 2010 | 9 | 10 | 8 | 9 | 10 | 4 | 6 | 3 | 3 | 8 | 10 | 8 |
| 2011 | 10 | 7 | 3 | 6 | 4 | 8 | 9 | 9 | 7 | 7 | 6 | 9 |
| 2012 | 8 | 4 | 5 | 6 | 10 | 9 | 8 | 8 | 6 | 9 | 11 | 6 |
| 2013 | 14 | 6 | 8 | 9 | 9 | 7 | 10 | 10 | 7 | 6 | 8 | 12 |
| 2014 | 3 | 7 | 8 | 6 | 5 | 7 | 11 | 6 | 10 | 6 | 9 | 6 |
| 2015 | 9 | 9 | 8 | 9 | 8 | 6 | 3 | 4 | 5 | 3 | 7 | 5 |
| 2016 | 8 | 9 | 6 | 11 | 9 | 12 | 5 | 4 | 7 | 4 | 4 | 5 |
| 2017 | 4 | 5 | 6 | 3 | 5 | 6 | 8 | 6 | 7 | 8 | 7 | 6 |
| 2018 | 6 | 7 | 4 | 11 | 7 | 10 | 4 | 5 | 3 | 4 | 6 | 5 |
| 2019 | 7 | 5 | 5 | 7 | 5 | 8 | 2 | 7 | 5 | 6 | 12 | 6 |
| 2020 | 6 | 3 | 2 | 5 | 2 | 3 | 2 | 8 | 5 | 6 | 6 | 5 |

Hidalgo

|  | Jan | Feb | Mar | Apr | May | Jun | Jul | Aug | Sep | Oct | Nov | Dec |
| --- | --- | --- | --- | --- | --- | --- | --- | --- | --- | --- | --- | --- |
| 2010 | 3 | 2 | 11 | 6 | 5 | 9 | 6 | 4 | 4 | 4 | 2 | 2 |
| 2011 | 6 | 13 | 13 | 15 | 10 | 12 | 8 | 9 | 8 | 9 | 9 | 9 |
| 2012 | 6 | 7 | 7 | 12 | 6 | 10 | 14 | 8 | 6 | 9 | 9 | 8 |
| 2013 | 6 | 6 | 11 | 10 | 14 | 14 | 16 | 10 | 9 | 8 | 6 | 9 |
| 2014 | 10 | 4 | 8 | 11 | 10 | 7 | 13 | 12 | 17 | 10 | 11 | 11 |
| 2015 | 10 | 15 | 11 | 13 | 11 | 10 | 7 | 11 | 2 | 13 | 8 | 7 |
| 2016 | 11 | 6 | 10 | 12 | 11 | 9 | 11 | 6 | 3 | 5 | 17 | 13 |
| 2017 | 11 | 4 | 11 | 9 | 13 | 14 | 13 | 15 | 5 | 8 | 6 | 6 |
| 2018 | 8 | 4 | 9 | 10 | 16 | 16 | 10 | 6 | 13 | 12 | 2 | 9 |
| 2019 | 8 | 9 | 8 | 14 | 16 | 4 | 8 | 13 | 13 | 10 | 9 | 10 |
| 2020 | 10 | 10 | 17 | 10 | 13 | 5 | 9 | 7 | 12 | 13 | 19 | 10 |

Jalisco

|  | Jan | Feb | Mar | Apr | May | Jun | Jul | Aug | Sep | Oct | Nov | Dec |
| --- | --- | --- | --- | --- | --- | --- | --- | --- | --- | --- | --- | --- |
| 2010 | 33 | 26 | 26 | 40 | 32 | 39 | 42 | 43 | 37 | 26 | 32 | 29 |
| 2011 | 36 | 39 | 32 | 44 | 38 | 43 | 36 | 47 | 28 | 40 | 42 | 25 |
| 2012 | 41 | 35 | 41 | 59 | 54 | 29 | 40 | 41 | 34 | 53 | 44 | 35 |
| 2013 | 34 | 37 | 46 | 45 | 60 | 42 | 42 | 60 | 28 | 31 | 34 | 34 |
| 2014 | 34 | 29 | 40 | 48 | 38 | 34 | 38 | 55 | 49 | 45 | 45 | 54 |
| 2015 | 42 | 32 | 53 | 33 | 38 | 46 | 36 | 54 | 41 | 44 | 52 | 58 |
| 2016 | 42 | 50 | 51 | 58 | 59 | 51 | 46 | 45 | 35 | 51 | 47 | 62 |
| 2017 | 54 | 60 | 59 | 61 | 51 | 57 | 58 | 80 | 67 | 41 | 40 | 46 |
| 2018 | 50 | 53 | 57 | 65 | 78 | 63 | 44 | 54 | 54 | 65 | 44 | 56 |
| 2019 | 48 | 48 | 40 | 66 | 50 | 48 | 52 | 57 | 56 | 53 | 47 | 60 |
| 2020 | 43 | 56 | 65 | 40 | 68 | 61 | 55 | 66 | 63 | 54 | 56 | 53 |

México

|  | Jan | Feb | Mar | Apr | May | Jun | Jul | Aug | Sep | Oct | Nov | Dec |
| --- | --- | --- | --- | --- | --- | --- | --- | --- | --- | --- | --- | --- |
| 2010 | 43 | 43 | 39 | 54 | 39 | 34 | 40 | 37 | 35 | 41 | 69 | 48 |
| 2011 | 49 | 37 | 64 | 63 | 57 | 41 | 41 | 45 | 52 | 52 | 38 | 48 |
| 2012 | 44 | 42 | 59 | 42 | 42 | 63 | 43 | 50 | 52 | 48 | 43 | 60 |
| 2013 | 60 | 40 | 41 | 51 | 57 | 46 | 62 | 45 | 42 | 62 | 45 | 53 |
| 2014 | 49 | 48 | 46 | 50 | 53 | 54 | 51 | 59 | 51 | 50 | 61 | 71 |
| 2015 | 50 | 47 | 57 | 60 | 42 | 72 | 58 | 48 | 54 | 49 | 63 | 54 |
| 2016 | 41 | 45 | 41 | 46 | 65 | 35 | 46 | 54 | 54 | 56 | 48 | 35 |
| 2017 | 54 | 48 | 34 | 39 | 68 | 46 | 38 | 43 | 49 | 45 | 44 | 49 |
| 2018 | 32 | 51 | 45 | 50 | 39 | 39 | 41 | 52 | 50 | 62 | 59 | 57 |
| 2019 | 58 | 46 | 63 | 58 | 64 | 58 | 55 | 63 | 62 | 43 | 75 | 66 |
| 2020 | 50 | 65 | 72 | 63 | 72 | 63 | 72 | 54 | 77 | 71 | 85 | 88 |

Michoacán

|  | Jan | Feb | Mar | Apr | May | Jun | Jul | Aug | Sep | Oct | Nov | Dec |
| --- | --- | --- | --- | --- | --- | --- | --- | --- | --- | --- | --- | --- |
| 2010 | 15 | 15 | 17 | 16 | 10 | 11 | 15 | 9 | 9 | 10 | 12 | 12 |
| 2011 | 12 | 16 | 14 | 12 | 17 | 18 | 8 | 17 | 8 | 16 | 10 | 8 |
| 2012 | 15 | 13 | 13 | 6 | 12 | 12 | 12 | 15 | 17 | 10 | 12 | 9 |
| 2013 | 19 | 9 | 10 | 9 | 11 | 9 | 10 | 16 | 9 | 9 | 12 | 17 |
| 2014 | 16 | 12 | 19 | 11 | 16 | 11 | 21 | 22 | 13 | 17 | 14 | 16 |
| 2015 | 8 | 13 | 11 | 14 | 15 | 13 | 18 | 18 | 17 | 16 | 9 | 13 |
| 2016 | 14 | 11 | 13 | 15 | 15 | 14 | 18 | 15 | 16 | 14 | 25 | 8 |
| 2017 | 11 | 12 | 16 | 14 | 27 | 16 | 12 | 17 | 15 | 22 | 22 | 18 |
| 2018 | 18 | 12 | 30 | 22 | 23 | 30 | 20 | 18 | 20 | 18 | 19 | 21 |
| 2019 | 26 | 25 | 29 | 27 | 32 | 28 | 28 | 20 | 34 | 28 | 26 | 32 |
| 2020 | 31 | 27 | 34 | 30 | 29 | 43 | 31 | 33 | 34 | 31 | 31 | 27 |

Morelos

|  | Jan | Feb | Mar | Apr | May | Jun | Jul | Aug | Sep | Oct | Nov | Dec |
| --- | --- | --- | --- | --- | --- | --- | --- | --- | --- | --- | --- | --- |
| 2010 | 1 | 6 | 6 | 3 | 6 | 4 | 1 | 6 | 3 | 3 | 3 | 6 |
| 2011 | 2 | 3 | 10 | 5 | 5 | 3 | 3 | 5 | 7 | 12 | 8 | 5 |
| 2012 | 6 | 4 | 3 | 6 | 5 | 7 | 5 | 7 | 4 | 4 | 4 | 9 |
| 2013 | 4 | 8 | 3 | 12 | 7 | 5 | 5 | 4 | 5 | 4 | 5 | 3 |
| 2014 | 1 | 6 | 5 | 7 | 4 | 7 | 6 | 6 | 3 | 3 | 4 | 1 |
| 2015 | 1 | 5 | 7 | 2 | 3 | 4 | 1 | 2 | 6 | 4 | 6 | 4 |
| 2016 | 4 | 5 | 9 | 12 | 8 | 7 | 9 | 10 | 7 | 9 | 5 | 5 |
| 2017 | 5 | 7 | 8 | 6 | 0 | 5 | 12 | 5 | 5 | 4 | 8 | 10 |
| 2018 | 4 | 7 | 6 | 5 | 11 | 8 | 15 | 4 | 5 | 12 | 7 | 10 |
| 2019 | 6 | 8 | 7 | 4 | 8 | 5 | 11 | 10 | 3 | 10 | 13 | 9 |
| 2020 | 14 | 7 | 7 | 14 | 9 | 4 | 14 | 11 | 7 | 5 | 10 | 12 |

Nayarit

|  | Jan | Feb | Mar | Apr | May | Jun | Jul | Aug | Sep | Oct | Nov | Dec |
| --- | --- | --- | --- | --- | --- | --- | --- | --- | --- | --- | --- | --- |
| 2010 | 5 | 8 | 2 | 3 | 1 | 3 | 6 | 2 | 3 | 3 | 4 | 5 |
| 2011 | 0 | 3 | 6 | 7 | 8 | 4 | 2 | 4 | 2 | 4 | 2 | 4 |
| 2012 | 2 | 6 | 1 | 5 | 1 | 2 | 4 | 6 | 7 | 5 | 6 | 5 |
| 2013 | 5 | 6 | 4 | 1 | 8 | 4 | 2 | 5 | 7 | 3 | 3 | 6 |
| 2014 | 7 | 2 | 9 | 6 | 6 | 3 | 5 | 7 | 7 | 11 | 12 | 5 |
| 2015 | 9 | 5 | 7 | 5 | 8 | 8 | 7 | 4 | 4 | 6 | 4 | 9 |
| 2016 | 7 | 6 | 4 | 6 | 3 | 8 | 7 | 11 | 9 | 10 | 6 | 10 |
| 2017 | 4 | 3 | 5 | 5 | 10 | 13 | 8 | 4 | 7 | 9 | 6 | 6 |
| 2018 | 9 | 6 | 1 | 7 | 11 | 8 | 8 | 6 | 1 | 7 | 4 | 7 |
| 2019 | 7 | 3 | 9 | 5 | 5 | 11 | 9 | 4 | 6 | 12 | 5 | 5 |
| 2020 | 7 | 3 | 16 | 13 | 9 | 7 | 6 | 7 | 9 | 4 | 5 | 7 |

Nuevo León

|  | Jan | Feb | Mar | Apr | May | Jun | Jul | Aug | Sep | Oct | Nov | Dec |
| --- | --- | --- | --- | --- | --- | --- | --- | --- | --- | --- | --- | --- |
| 2010 | 20 | 14 | 19 | 15 | 24 | 16 | 25 | 30 | 22 | 18 | 13 | 20 |
| 2011 | 25 | 12 | 15 | 13 | 21 | 15 | 19 | 23 | 17 | 21 | 24 | 17 |
| 2012 | 16 | 10 | 18 | 12 | 26 | 14 | 22 | 28 | 29 | 22 | 25 | 9 |
| 2013 | 14 | 16 | 15 | 20 | 22 | 23 | 30 | 22 | 26 | 26 | 18 | 15 |
| 2014 | 11 | 6 | 17 | 21 | 20 | 20 | 23 | 22 | 15 | 23 | 16 | 25 |
| 2015 | 15 | 16 | 25 | 28 | 16 | 27 | 25 | 20 | 21 | 18 | 19 | 26 |
| 2016 | 20 | 21 | 23 | 21 | 32 | 33 | 20 | 29 | 29 | 36 | 13 | 15 |
| 2017 | 18 | 23 | 22 | 24 | 23 | 31 | 24 | 26 | 18 | 17 | 27 | 16 |
| 2018 | 18 | 36 | 35 | 36 | 30 | 20 | 32 | 34 | 28 | 27 | 14 | 23 |
| 2019 | 20 | 21 | 26 | 29 | 30 | 25 | 29 | 43 | 27 | 33 | 24 | 23 |
| 2020 | 35 | 16 | 41 | 15 | 28 | 33 | 35 | 36 | 38 | 37 | 26 | 27 |

Oaxaca

|  | Jan | Feb | Mar | Apr | May | Jun | Jul | Aug | Sep | Oct | Nov | Dec |
| --- | --- | --- | --- | --- | --- | --- | --- | --- | --- | --- | --- | --- |
| 2010 | 11 | 12 | 10 | 12 | 16 | 13 | 10 | 16 | 8 | 9 | 6 | 8 |
| 2011 | 6 | 10 | 12 | 9 | 18 | 15 | 6 | 18 | 14 | 10 | 10 | 13 |
| 2012 | 11 | 4 | 17 | 5 | 16 | 10 | 12 | 6 | 8 | 13 | 10 | 12 |
| 2013 | 7 | 8 | 8 | 13 | 13 | 12 | 14 | 12 | 6 | 9 | 10 | 10 |
| 2014 | 11 | 9 | 15 | 12 | 16 | 12 | 17 | 6 | 11 | 11 | 4 | 6 |
| 2015 | 17 | 16 | 12 | 17 | 6 | 8 | 11 | 19 | 14 | 13 | 13 | 13 |
| 2016 | 11 | 10 | 11 | 10 | 12 | 6 | 6 | 9 | 8 | 9 | 8 | 16 |
| 2017 | 13 | 8 | 12 | 16 | 12 | 9 | 6 | 17 | 7 | 9 | 9 | 11 |
| 2018 | 8 | 10 | 11 | 23 | 9 | 19 | 13 | 14 | 14 | 13 | 10 | 4 |
| 2019 | 25 | 10 | 10 | 13 | 13 | 13 | 20 | 10 | 14 | 9 | 15 | 13 |
| 2020 | 20 | 11 | 14 | 23 | 23 | 13 | 25 | 13 | 18 | 17 | 18 | 15 |

Puebla

|  | Jan | Feb | Mar | Apr | May | Jun | Jul | Aug | Sep | Oct | Nov | Dec |
| --- | --- | --- | --- | --- | --- | --- | --- | --- | --- | --- | --- | --- |
| 2010 | 14 | 24 | 21 | 16 | 13 | 17 | 13 | 13 | 21 | 17 | 19 | 15 |
| 2011 | 26 | 22 | 22 | 22 | 12 | 13 | 12 | 23 | 24 | 22 | 20 | 32 |
| 2012 | 17 | 10 | 15 | 15 | 19 | 21 | 14 | 22 | 22 | 13 | 20 | 27 |
| 2013 | 17 | 24 | 21 | 21 | 22 | 16 | 18 | 23 | 17 | 23 | 16 | 19 |
| 2014 | 13 | 17 | 23 | 19 | 22 | 31 | 22 | 29 | 22 | 29 | 27 | 21 |
| 2015 | 25 | 23 | 23 | 17 | 29 | 27 | 21 | 18 | 19 | 18 | 25 | 25 |
| 2016 | 17 | 19 | 21 | 27 | 27 | 20 | 19 | 15 | 16 | 15 | 15 | 17 |
| 2017 | 29 | 20 | 30 | 24 | 32 | 29 | 26 | 21 | 22 | 20 | 22 | 30 |
| 2018 | 25 | 27 | 26 | 26 | 36 | 35 | 29 | 30 | 21 | 24 | 29 | 30 |
| 2019 | 26 | 21 | 30 | 22 | 43 | 30 | 22 | 26 | 22 | 36 | 36 | 33 |
| 2020 | 27 | 30 | 31 | 23 | 44 | 34 | 26 | 24 | 23 | 31 | 23 | 33 |

Querétaro

|  | Jan | Feb | Mar | Apr | May | Jun | Jul | Aug | Sep | Oct | Nov | Dec |
| --- | --- | --- | --- | --- | --- | --- | --- | --- | --- | --- | --- | --- |
| 2010 | 5 | 9 | 4 | 7 | 8 | 4 | 14 | 5 | 10 | 10 | 5 | 5 |
| 2011 | 7 | 7 | 6 | 8 | 11 | 6 | 9 | 9 | 9 | 5 | 14 | 10 |
| 2012 | 7 | 10 | 11 | 13 | 11 | 7 | 12 | 8 | 8 | 9 | 7 | 13 |
| 2013 | 5 | 5 | 15 | 9 | 11 | 13 | 6 | 12 | 7 | 12 | 8 | 7 |
| 2014 | 5 | 7 | 4 | 3 | 4 | 9 | 9 | 11 | 9 | 5 | 6 | 7 |
| 2015 | 8 | 10 | 8 | 12 | 8 | 10 | 10 | 4 | 8 | 13 | 10 | 10 |
| 2016 | 10 | 7 | 5 | 12 | 12 | 10 | 6 | 14 | 12 | 5 | 8 | 5 |
| 2017 | 9 | 9 | 10 | 11 | 12 | 15 | 10 | 19 | 12 | 9 | 10 | 17 |
| 2018 | 7 | 10 | 11 | 18 | 14 | 10 | 7 | 9 | 8 | 14 | 13 | 18 |
| 2019 | 9 | 11 | 14 | 18 | 11 | 12 | 18 | 27 | 18 | 13 | 21 | 12 |
| 2020 | 9 | 20 | 24 | 17 | 14 | 19 | 11 | 17 | 18 | 18 | 20 | 14 |

Quintana Roo

|  | Jan | Feb | Mar | Apr | May | Jun | Jul | Aug | Sep | Oct | Nov | Dec |
| --- | --- | --- | --- | --- | --- | --- | --- | --- | --- | --- | --- | --- |
| 2010 | 16 | 9 | 15 | 11 | 20 | 10 | 9 | 9 | 6 | 11 | 8 | 9 |
| 2011 | 12 | 7 | 6 | 11 | 13 | 11 | 9 | 15 | 14 | 7 | 14 | 5 |
| 2012 | 11 | 13 | 6 | 10 | 19 | 15 | 18 | 9 | 12 | 12 | 11 | 9 |
| 2013 | 18 | 12 | 12 | 7 | 11 | 13 | 9 | 21 | 15 | 10 | 9 | 6 |
| 2014 | 7 | 13 | 11 | 8 | 10 | 12 | 10 | 13 | 8 | 10 | 7 | 12 |
| 2015 | 6 | 16 | 14 | 19 | 9 | 11 | 13 | 14 | 14 | 4 | 14 | 14 |
| 2016 | 7 | 9 | 9 | 14 | 10 | 7 | 10 | 13 | 10 | 16 | 13 | 16 |
| 2017 | 10 | 12 | 9 | 16 | 11 | 11 | 11 | 11 | 18 | 10 | 8 | 10 |
| 2018 | 8 | 9 | 11 | 7 | 12 | 6 | 16 | 14 | 10 | 17 | 12 | 9 |
| 2019 | 13 | 5 | 11 | 14 | 18 | 11 | 14 | 10 | 8 | 10 | 11 | 12 |
| 2020 | 9 | 15 | 22 | 16 | 9 | 16 | 16 | 17 | 15 | 13 | 17 | 14 |

San Luis Potosí

|  | Jan | Feb | Mar | Apr | May | Jun | Jul | Aug | Sep | Oct | Nov | Dec |
| --- | --- | --- | --- | --- | --- | --- | --- | --- | --- | --- | --- | --- |
| 2010 | 14 | 10 | 11 | 10 | 16 | 28 | 9 | 13 | 15 | 13 | 15 | 11 |
| 2011 | 8 | 11 | 17 | 15 | 22 | 13 | 5 | 7 | 20 | 12 | 18 | 10 |
| 2012 | 9 | 14 | 6 | 12 | 17 | 16 | 18 | 19 | 15 | 15 | 10 | 10 |
| 2013 | 23 | 13 | 11 | 22 | 10 | 10 | 10 | 13 | 11 | 6 | 13 | 14 |
| 2014 | 23 | 14 | 13 | 17 | 17 | 10 | 19 | 9 | 14 | 24 | 5 | 12 |
| 2015 | 12 | 14 | 19 | 12 | 19 | 15 | 10 | 12 | 10 | 13 | 11 | 18 |
| 2016 | 17 | 14 | 17 | 18 | 14 | 11 | 10 | 13 | 18 | 17 | 14 | 16 |
| 2017 | 12 | 18 | 16 | 24 | 14 | 16 | 13 | 19 | 23 | 14 | 17 | 11 |
| 2018 | 20 | 14 | 30 | 13 | 22 | 12 | 15 | 12 | 21 | 21 | 15 | 15 |
| 2019 | 20 | 12 | 14 | 12 | 26 | 24 | 23 | 24 | 26 | 24 | 19 | 19 |
| 2020 | 21 | 21 | 22 | 19 | 17 | 19 | 32 | 19 | 17 | 29 | 26 | 20 |

Sinaloa

|  | Jan | Feb | Mar | Apr | May | Jun | Jul | Aug | Sep | Oct | Nov | Dec |
| --- | --- | --- | --- | --- | --- | --- | --- | --- | --- | --- | --- | --- |
| 2010 | 7 | 9 | 12 | 8 | 9 | 10 | 15 | 8 | 6 | 17 | 8 | 2 |
| 2011 | 4 | 9 | 12 | 9 | 5 | 6 | 12 | 11 | 7 | 10 | 15 | 11 |
| 2012 | 6 | 12 | 10 | 8 | 5 | 10 | 9 | 6 | 8 | 7 | 12 | 9 |
| 2013 | 10 | 8 | 16 | 12 | 12 | 11 | 6 | 7 | 7 | 8 | 10 | 15 |
| 2014 | 16 | 10 | 10 | 11 | 10 | 10 | 10 | 12 | 18 | 12 | 8 | 20 |
| 2015 | 13 | 9 | 9 | 7 | 9 | 14 | 11 | 21 | 14 | 13 | 12 | 12 |
| 2016 | 9 | 13 | 13 | 12 | 8 | 19 | 15 | 11 | 18 | 20 | 4 | 23 |
| 2017 | 13 | 14 | 15 | 11 | 15 | 13 | 21 | 11 | 16 | 7 | 14 | 17 |
| 2018 | 11 | 4 | 14 | 10 | 16 | 16 | 10 | 13 | 17 | 16 | 15 | 17 |
| 2019 | 8 | 16 | 14 | 18 | 10 | 9 | 13 | 15 | 16 | 11 | 11 | 13 |
| 2020 | 15 | 10 | 6 | 9 | 11 | 10 | 8 | 17 | 7 | 14 | 6 | 8 |

Sonora

|  | Jan | Feb | Mar | Apr | May | Jun | Jul | Aug | Sep | Oct | Nov | Dec |
| --- | --- | --- | --- | --- | --- | --- | --- | --- | --- | --- | --- | --- |
| 2010 | 15 | 12 | 14 | 14 | 14 | 24 | 10 | 10 | 24 | 13 | 11 | 10 |
| 2011 | 12 | 9 | 13 | 9 | 19 | 22 | 11 | 16 | 18 | 18 | 12 | 22 |
| 2012 | 22 | 12 | 17 | 16 | 23 | 12 | 10 | 17 | 10 | 18 | 16 | 12 |
| 2013 | 16 | 9 | 25 | 22 | 20 | 24 | 24 | 26 | 25 | 17 | 18 | 17 |
| 2014 | 19 | 19 | 24 | 26 | 19 | 20 | 22 | 15 | 20 | 16 | 13 | 28 |
| 2015 | 20 | 12 | 23 | 22 | 30 | 24 | 19 | 21 | 22 | 24 | 16 | 18 |
| 2016 | 17 | 15 | 18 | 24 | 13 | 19 | 21 | 23 | 19 | 22 | 11 | 21 |
| 2017 | 27 | 17 | 21 | 22 | 29 | 20 | 18 | 31 | 23 | 22 | 27 | 19 |
| 2018 | 14 | 23 | 20 | 29 | 23 | 40 | 29 | 30 | 17 | 17 | 21 | 23 |
| 2019 | 18 | 14 | 29 | 26 | 34 | 20 | 22 | 24 | 29 | 27 | 18 | 14 |
| 2020 | 31 | 14 | 22 | 25 | 24 | 31 | 32 | 25 | 35 | 27 | 27 | 26 |

Tabasco

|  | Jan | Feb | Mar | Apr | May | Jun | Jul | Aug | Sep | Oct | Nov | Dec |
| --- | --- | --- | --- | --- | --- | --- | --- | --- | --- | --- | --- | --- |
| 2010 | 11 | 10 | 20 | 15 | 15 | 15 | 28 | 20 | 15 | 7 | 13 | 21 |
| 2011 | 19 | 12 | 20 | 23 | 17 | 16 | 18 | 21 | 15 | 11 | 10 | 9 |
| 2012 | 12 | 7 | 24 | 18 | 12 | 18 | 14 | 12 | 15 | 19 | 16 | 9 |
| 2013 | 12 | 14 | 9 | 25 | 12 | 16 | 15 | 21 | 16 | 12 | 7 | 10 |
| 2014 | 5 | 15 | 24 | 9 | 18 | 14 | 16 | 20 | 13 | 12 | 12 | 10 |
| 2015 | 10 | 8 | 21 | 12 | 15 | 18 | 23 | 16 | 10 | 18 | 12 | 10 |
| 2016 | 11 | 9 | 17 | 20 | 16 | 10 | 19 | 9 | 12 | 12 | 11 | 11 |
| 2017 | 8 | 12 | 6 | 13 | 10 | 9 | 6 | 8 | 10 | 11 | 6 | 9 |
| 2018 | 13 | 5 | 13 | 12 | 15 | 10 | 22 | 10 | 9 | 11 | 14 | 15 |
| 2019 | 13 | 9 | 16 | 10 | 13 | 9 | 10 | 17 | 7 | 8 | 7 | 11 |
| 2020 | 13 | 6 | 13 | 1 | 4 | 5 | 16 | 19 | 18 | 12 | 6 | 6 |

Tamaulipas

|  | Jan | Feb | Mar | Apr | May | Jun | Jul | Aug | Sep | Oct | Nov | Dec |
| --- | --- | --- | --- | --- | --- | --- | --- | --- | --- | --- | --- | --- |
| 2010 | 12 | 14 | 12 | 18 | 18 | 12 | 8 | 20 | 15 | 13 | 17 | 9 |
| 2011 | 9 | 15 | 14 | 7 | 17 | 15 | 17 | 11 | 11 | 16 | 12 | 8 |
| 2012 | 15 | 14 | 16 | 20 | 15 | 17 | 13 | 12 | 15 | 8 | 11 | 13 |
| 2013 | 7 | 13 | 11 | 20 | 13 | 21 | 17 | 14 | 13 | 9 | 12 | 12 |
| 2014 | 13 | 9 | 18 | 21 | 21 | 11 | 11 | 14 | 18 | 12 | 15 | 17 |
| 2015 | 8 | 10 | 7 | 15 | 15 | 16 | 18 | 21 | 13 | 9 | 6 | 11 |
| 2016 | 8 | 13 | 16 | 15 | 14 | 13 | 18 | 22 | 14 | 10 | 11 | 9 |
| 2017 | 12 | 8 | 16 | 12 | 21 | 24 | 12 | 22 | 13 | 12 | 7 | 12 |
| 2018 | 17 | 9 | 9 | 5 | 18 | 24 | 23 | 16 | 17 | 12 | 11 | 14 |
| 2019 | 13 | 9 | 7 | 20 | 23 | 16 | 19 | 21 | 13 | 13 | 19 | 12 |
| 2020 | 17 | 11 | 12 | 16 | 14 | 16 | 21 | 21 | 14 | 9 | 15 | 12 |

Tlaxcala

|  | Jan | Feb | Mar | Apr | May | Jun | Jul | Aug | Sep | Oct | Nov | Dec |
| --- | --- | --- | --- | --- | --- | --- | --- | --- | --- | --- | --- | --- |
| 2010 | 3 | 5 | 1 | 1 | 7 | 2 | 1 | 3 | 3 | 1 | 4 | 1 |
| 2011 | 5 | 4 | 3 | 4 | 7 | 2 | 7 | 7 | 2 | 5 | 4 | 2 |
| 2012 | 2 | 4 | 2 | 3 | 3 | 6 | 2 | 2 | 6 | 4 | 3 | 6 |
| 2013 | 3 | 3 | 5 | 0 | 3 | 2 | 8 | 2 | 3 | 5 | 4 | 5 |
| 2014 | 4 | 5 | 6 | 3 | 4 | 4 | 6 | 1 | 6 | 5 | 6 | 5 |
| 2015 | 3 | 2 | 3 | 5 | 6 | 6 | 2 | 5 | 8 | 5 | 3 | 3 |
| 2016 | 2 | 7 | 4 | 9 | 3 | 4 | 2 | 3 | 4 | 8 | 4 | 3 |
| 2017 | 7 | 4 | 4 | 7 | 7 | 3 | 6 | 3 | 3 | 6 | 3 | 2 |
| 2018 | 2 | 3 | 3 | 4 | 4 | 5 | 4 | 5 | 6 | 7 | 7 | 3 |
| 2019 | 7 | 4 | 6 | 5 | 4 | 6 | 11 | 6 | 3 | 7 | 1 | 2 |
| 2020 | 10 | 7 | 2 | 4 | 6 | 2 | 2 | 6 | 4 | 8 | 2 | 3 |

Veracruz

|  | Jan | Feb | Mar | Apr | May | Jun | Jul | Aug | Sep | Oct | Nov | Dec |
| --- | --- | --- | --- | --- | --- | --- | --- | --- | --- | --- | --- | --- |
| 2010 | 16 | 20 | 23 | 23 | 26 | 34 | 22 | 19 | 35 | 11 | 25 | 10 |
| 2011 | 28 | 21 | 26 | 31 | 27 | 23 | 27 | 28 | 27 | 31 | 22 | 15 |
| 2012 | 31 | 25 | 22 | 29 | 24 | 27 | 23 | 23 | 25 | 18 | 20 | 28 |
| 2013 | 21 | 20 | 24 | 19 | 23 | 25 | 25 | 23 | 25 | 24 | 21 | 12 |
| 2014 | 31 | 23 | 35 | 24 | 24 | 40 | 29 | 26 | 23 | 27 | 16 | 27 |
| 2015 | 25 | 18 | 20 | 33 | 20 | 25 | 21 | 18 | 12 | 22 | 18 | 17 |
| 2016 | 12 | 12 | 11 | 15 | 17 | 26 | 31 | 19 | 19 | 14 | 13 | 14 |
| 2017 | 15 | 12 | 24 | 19 | 24 | 19 | 21 | 14 | 18 | 18 | 15 | 20 |
| 2018 | 19 | 20 | 18 | 26 | 23 | 21 | 25 | 21 | 19 | 20 | 20 | 16 |
| 2019 | 24 | 19 | 18 | 28 | 29 | 28 | 25 | 22 | 19 | 16 | 19 | 17 |
| 2020 | 26 | 20 | 29 | 22 | 22 | 10 | 37 | 28 | 26 | 21 | 21 | 21 |

Yucatán

|  | Jan | Feb | Mar | Apr | May | Jun | Jul | Aug | Sep | Oct | Nov | Dec |
| --- | --- | --- | --- | --- | --- | --- | --- | --- | --- | --- | --- | --- |
| 2010 | 15 | 11 | 28 | 24 | 20 | 17 | 10 | 23 | 20 | 12 | 20 | 9 |
| 2011 | 10 | 19 | 21 | 14 | 17 | 12 | 25 | 13 | 16 | 15 | 14 | 10 |
| 2012 | 14 | 13 | 18 | 14 | 8 | 14 | 13 | 15 | 13 | 18 | 9 | 10 |
| 2013 | 8 | 12 | 14 | 14 | 24 | 16 | 18 | 16 | 15 | 12 | 9 | 14 |
| 2014 | 8 | 16 | 14 | 10 | 14 | 13 | 20 | 23 | 21 | 17 | 18 | 8 |
| 2015 | 15 | 7 | 14 | 12 | 21 | 18 | 19 | 25 | 18 | 14 | 10 | 14 |
| 2016 | 18 | 14 | 17 | 17 | 24 | 18 | 31 | 18 | 15 | 14 | 16 | 21 |
| 2017 | 18 | 10 | 16 | 15 | 22 | 16 | 22 | 17 | 13 | 13 | 16 | 18 |
| 2018 | 11 | 26 | 24 | 22 | 26 | 21 | 20 | 18 | 20 | 18 | 19 | 17 |
| 2019 | 17 | 17 | 23 | 24 | 18 | 19 | 22 | 28 | 26 | 19 | 19 | 15 |
| 2020 | 21 | 17 | 26 | 19 | 12 | 19 | 19 | 22 | 27 | 27 | 18 | 19 |

Zacatecas

|  | Jan | Feb | Mar | Apr | May | Jun | Jul | Aug | Sep | Oct | Nov | Dec |
| --- | --- | --- | --- | --- | --- | --- | --- | --- | --- | --- | --- | --- |
| 2010 | 4 | 2 | 5 | 8 | 8 | 5 | 4 | 3 | 4 | 6 | 1 | 2 |
| 2011 | 6 | 5 | 5 | 8 | 16 | 5 | 2 | 11 | 4 | 6 | 8 | 7 |
| 2012 | 4 | 5 | 7 | 6 | 4 | 4 | 6 | 4 | 4 | 6 | 4 | 2 |
| 2013 | 7 | 8 | 4 | 11 | 4 | 3 | 7 | 3 | 4 | 4 | 0 | 4 |
| 2014 | 5 | 4 | 6 | 5 | 6 | 8 | 6 | 7 | 7 | 3 | 9 | 4 |
| 2015 | 9 | 10 | 9 | 10 | 8 | 4 | 11 | 15 | 4 | 9 | 9 | 7 |
| 2016 | 9 | 4 | 5 | 8 | 9 | 6 | 4 | 7 | 3 | 8 | 2 | 9 |
| 2017 | 11 | 9 | 12 | 7 | 11 | 11 | 5 | 9 | 8 | 13 | 6 | 9 |
| 2018 | 16 | 6 | 9 | 6 | 15 | 9 | 7 | 10 | 6 | 5 | 9 | 3 |
| 2019 | 6 | 14 | 13 | 15 | 10 | 6 | 6 | 8 | 9 | 9 | 12 | 10 |
| 2020 | 9 | 9 | 11 | 7 | 13 | 17 | 10 | 9 | 14 | 13 | 11 | 6 |
